# Predictors for Return to Work in Subjects with Mild Traumatic Brain Injury

**DOI:** 10.1155/2016/8026414

**Published:** 2016-02-14

**Authors:** Eirik Vikane, Torgeir Hellstrøm, Cecilie Røe, Erik Bautz-Holter, Jörg Aßmus, Jan Sture Skouen

**Affiliations:** ^1^Department of Physical Medicine and Rehabilitation, Haukeland University Hospital, 5021 Bergen, Norway; ^2^Department of Global Public Health and Primary Care, University of Bergen, 5020 Bergen, Norway; ^3^Department of Physical Medicine and Rehabilitation, Oslo University Hospital, 0424 Oslo, Norway; ^4^Faculty of Medicine, University of Oslo, 0316 Oslo, Norway; ^5^Centre for Clinical Research, Haukeland University Hospital, 5021 Bergen, Norway

## Abstract

*Objective*. To predict return to work (RTW) at 12 months for patients who either were sick-listed or were at risk to be sick-listed with persistent postconcussion symptoms (PCS) at six to eight weeks after injury.* Method*. A prospective cohort study of 151 patients with mild traumatic brain injury (MTBI) admitted consecutively to outpatient clinics at two University Hospitals in Norway. The study was conducted as part of a randomised clinical trial. Injury characteristics were obtained from the medical records. Sick leave data from one year before to one year after MTBI were obtained from the Norwegian Labour and Welfare Service. Self-report questionnaires were used to obtain demographic and symptom profiles.* Results*. We observed a significant negative association between RTW at 12 months and psychological distress, global functioning, and being sick-listed at two months after MTBI, as well as having been sick-listed the last year before injury.* Conclusion*. Psychological distress, global functioning postinjury, and the sick leave trajectory of the subjects were negative predictors for RTW. These findings should be taken into consideration when evaluating future vocational rehabilitation models.

## 1. Introduction

The majority of patients suffering a head injury sustain a mild traumatic brain injury (MTBI) [1]. The true population-based incidence for MTBI is likely more than 600 patients per 100 000 people, and, as such, it is a major public-health concern [1, 2]. A systematic review concluded that most workers return to work (RTW) within three to six months after MTBI, with approximately 5% to 20% facing persistent problems [3]. The existing literature demonstrates that RTW after one year varies from approximately 42% to 97%, likely due to varying patient characteristics, geographic regions, occupational categories, compensation systems, and definition of MTBI [3–8]. To be unemployed affects several dimensions of physical, psychological, and social health [8–10]. RTW and vocational status represent one of the best indicators of real world functioning [11]. Identifying predictors for delayed RTW may help to identify those who may benefit from a follow-up rehabilitation program [3, 12–14].

Several authors underline the need for a greater focus on the management of persistent postconcussion symptoms (PCS) to improve RTW [15, 16]. However, the majority of patients who return to work still present symptoms, and recently published reviews found different predictors for functional recovery in general as compared to RTW [3, 17, 18]. Preinjury variables, such as education, occupational factors (job independence and decision-making latitude), and age, are well-documented predictors for RTW [3, 4, 8, 12, 19].

Injury-related factors for RTW include multiple bodily injuries and intracranial abnormalities, where the associations between RTW and intracranial computed tomography abnormalities are inconsistent [3, 8, 13]. Nolin and Heroux concluded that patient characteristics, injury severity indicators, and cognitive functions postinjury were not associated with vocational status 12 to 36 months after MTBI. Only the total numbers of symptoms reported at follow-up 12 to 36 months after MTBI were related to vocational status at follow-up [20]. Other postinjury predictors for RTW are nausea or vomiting on hospital admission, severe pain early after injury, fatigue, dizziness, number of subjective symptoms, cognitive variables, financial compensation-seeking, and environmental factors such as social interaction [3, 4, 8, 12, 19, 21, 22]. Among several postinjury factors, headache and widespread pain are a common complication after MTBI [23–26]. Acute headache and pain after MTBI affect quality of life and daily function, with potential long-term effects on cognition, mood, sleep, and PCS [25]. However, to the best of our knowledge, little is known about the association between RTW and long lasting pain after MTBI.

Several authors emphasise that patients with persistent symptoms may differ from the majority of patients with MTBI who recover within three months [12, 20, 27]. Therefore, we must better identify which factors are predictive for RTW for patients with remaining symptoms a few months after injury. In a clinical setting, it is important to know which variables are likely to predict the outcome at follow-up consultations, and there is a need for better screening batteries based on predictors, which can be easily administered to the patients in the subacute stage. The optimal timing to evaluate the outcome after MTBI is not clear. The majority of workers return to work within six months after MTBI. Cancelliere et al. concluded that further research is needed to determine long-term RTW more than two years after MTBI, and Stulemeijer et al. concluded that six months is too early to determine final outcome after MTBI because many patients are in the process of rehabilitation [3, 13]. Gjesdal et al. found that absence from work beyond 20 weeks was a predictor of disability pension among persons who had long-term sickness absence in Norway [28]. It is therefore import to focus on early RTW after an injury to avoid forced retirement, and we therefore addressed the outcome RTW at 12 months after MTBI in this study.

The objective of this study was to identify which clinical characteristics predict RTW at 12 months for patients who were either sick-listed or at risk to be sick-listed with persistent PCS six to eight weeks after MTBI.

## 2. Methods

### 2.1. Study Design

This was a prospective cohort study of patients with MTBI known to have persistent symptoms. The study was conducted as part of a randomised clinical trial (RCT). The effect of the intervention is prepared as a separate submitted manuscript. Preinjury, injury-related, and postinjury clinical variables presented at six to eight weeks after MTBI together with some relevant clinical data from the emergency stay at hospital were used to find any significant associations with RTW 12 months after MTBI.

### 2.2. Participants

Adult patients aged 16–55 years who were hospitalised acutely at the Department of Neurosurgery for MTBI and who were either sick-listed or at risk to be sick-listed with persistent PCS six to eight weeks after injury were consecutively recruited to the study. MTBI was defined using the criteria from the Task Force on MTBI and the American Congress of Rehabilitation Medicine, defined as a Glasgow Coma Scale (GCS) measure of 13–15 within 30 minutes or the lowest score during the first 24 hours after injury, unconsciousness for less than 30 minutes, and posttraumatic amnesia for less than 24 hours [29, 30].

*Exclusion criteria* included current major psychiatric disease, major head trauma, or other diseases that had a significant impact on working skills, unemployment in the last six months, lack of Norwegian language skills, diagnosis with substance abuse problems given in the medical records, or lack of informed consent.

### 2.3. Study Settings

Patients hospitalised acutely after a trauma at the Department of Neurosurgery, with an ICD-10 diagnosis of S06.0–S06.9, were offered a planned clinical follow-up at an outpatient clinic at the Department of Physical Medicine and Rehabilitation at Haukeland University Hospital, in Bergen, and Oslo University Hospital, Oslo, Norway, from March 2009 to February 2012. The population was restricted to inhabitants of Hordaland, Oslo, and Akershus County including the cities of Bergen and Oslo, respectively, a mixed rural and urban community where the majority of the inhabitants are Norwegian residents (Caucasians).

### 2.4. Procedures

When potential participants were discharged from the emergency hospital, they received a self-report questionnaire by mail and an appointment by a specialist in physical and rehabilitation medicine at an outpatient clinic six to eight weeks after injury.

The questionnaire screened for postconcussion symptoms (PCS), psychological distress, disability, and pain.

The rehabilitation specialist conducted a clinical interview and a clinical examination with reassurance of an expected favourable outcome after the injury. Patients meeting the inclusion criteria were then offered to participate in the study and to participate in an additional RCT. The participants received a multidisciplinary examination and were then randomised into two groups; they were either randomised to a multidisciplinary outpatient treatment (intervention group) or referred back to their general practitioner with good advices and directions for further treatment if needed (control group). The multidisciplinary outpatient treatment consisted of individual consultations and a psychoeducational group intervention for four days over a period of four weeks. The participants shared experiences and received education about common problems after MTBI including topics related to RTW. Individualised treatment and clinical follow-ups in the first year were provided as needed.

### 2.5. Measures

RTW 12 months after injury was used as the main outcome and was the dependent variable. Data regarding sick leave one year before and the first year after the injury were collected from a national register, the Norwegian Labour and Welfare Service (NAV). Regardless of the diagnosis, the subjects were categorised as sick-listed or not.

Preinjury factors, injury-related factors, and postinjury factors were examined as potential predictors for RTW.

#### 2.5.1. Preinjury Factors

Preinjury factors assessed from the questionnaire consisted of age in years, sex, relationship status, number of children still living with parents, education, and employment status. Education was categorised as lower or higher education (13 years or more of formal education).

The self-report questionnaire contained information about smoking habits, alcohol consumption, and earlier diseases such as anxiety, depression, prior head injury, headache, and neurological disease, as well as other diseases. In the final analysis, we used information about earlier sick leave from NAV.

#### 2.5.2. Injury-Related Factors

Injury mechanisms classified as traffic accidents, falls, violence, and others (sports) were collected from the questionnaire. Occupational injuries were also registered in the questionnaire. The Glasgow Coma Scale (GCS) score, neurological status, headache, neck pain, findings on CT scan, alcohol intoxication, and length of hospital stay were registered during the emergency stay and were obtained from the medical record. GCS was used to classify MTBI. GCS was assessed to indicate the depth and duration of unconsciousness within 30 minutes or, subsequently, the score over the first 24 hours [31].

In the preliminary analyses, findings on CT were categorised as type of bleeding, contusion, location of injury, and fractures of the skull, face, and neck. In the final analyses, we either used intracranial injury or not. Length of posttraumatic amnesia (PTA) was based on both the medical records and the clinical interview six to eight weeks after injury, during which the patients were asked to recall events retrospectively.

#### 2.5.3. Postinjury Factors Were Collected Six to Eight Weeks after MTBI

Postconcussion symptoms (PCS) were measured using the Rivermead Post-Concussion Symptoms Questionnaire (RPQ). In RPQ, the patients are asked to rate the degree to which 16 items of the most frequently reported TBI-related symptoms are a problem compared with preinjury levels. The degree of the problem is rated on a 5-point Likert scale: 0 = not experienced at all, 1 = no longer a problem, 2 = a mild problem, 3 = a moderate problem, and 4 = a severe problem [32]. RPQ is documented to have high reliability for PCS, yet lacking good validity, and several authors argue against using the total sum score as recommended by King et al. [32–34]. Other authors have used the number of symptoms or a symptom by symptom comparison as an outcome [12, 20, 34]. Hence, we counted the total number of complaints with a RPQ score ≥ 2 six to eight weeks after injury when analysing predictors for RTW at 12 months.

Posttraumatic Stress Syndrome 10-Questions Inventory (PTSS-10) is a patient-reported inventory where 10 single items specific for posttraumatic stress disorder are rated from 1 to 7: 1 = never and 7 = always. PTSS-10 is found to be reliable and valid for screening out psychiatric risk cases among traumatized subjects [35–37]. In our analysis, we used the total score for PTSS-10.

Hospital Anxiety and Depression Scale (HAD) consists of 14 items detecting states of depression (7 items) and anxiety (7 items), rated on a 4-point scale from 0 to 3: 0 = no symptoms and 3 = a severe symptom or symptoms most of the time. It is validated for traumatic brain injuries (TBI) and documented to have high reliability [38, 39]. The total sum of scores for HAD was used in the analyses.

The subjective health complaints (SHC) questionnaire is a generic questionnaire that consists of 29 questions concerning severity and duration of subjective somatic and psychological complaints rated from 0 to 3: 0 = not at all, 1 = a little, 2 = some, and 3 = serious problems. The SHC is validated and is reliable for scoring subjective health complaints. The total number of complaints at SHC was used in our analysis [40, 41].

The numerical rating scale (NRS) registers pain in the head, pain in the neck and shoulders, and pain in the back and legs, rating pain from 0, which is no pain, to 10, which is pain as bad as it can be [42]. The NRS is reliable, easy, and commonly used measure for pain [43]. In our preliminary analyses, we used the NRS for pain in the head, the neck, and the back, and in addition we used both the total score for the three items and the highest score of the three items as a single item.

A pain drawing registered the location and numbers of areas affected by pain (rated from having 0 to 10 areas), where higher scores indicated widespread pain [44].

Glasgow Outcome Scale Extended (GOSE) is an ordinal, global 8-point scale for assessment of function within the areas of independence, work, social and leisure activities, and participation in social life. GOSE is a reliable and valid outcome measure widely used after TBI [45, 46]. Before inclusion in the study, a physician scored GOSE at baseline six to eight weeks after MTBI. In the final analyses, the categories were divided into good recovery (GOSE = 7 or 8), moderate disability (GOSE = 6), and severe and moderate disability (GOSE = 5 or less).

In the questionnaire, we asked if the patients had an expectation of a favourable outcome. If they answered yes or were recovered, they were classified as having a favourable expectation. If they answered no or did not know the outcome, they were classified as having a negative expectation of the outcome [47].

Data registered in the study were entered by two independent persons unfamiliar with the aim and content of the study. A statistician who did not participate in the treatment was responsible for the statistical analyses and controlled the analyses in instances where it was performed by the first author.

### 2.6. Statistical Methods

Data analyses were completed with IBM SPSS Statistics for Windows, Version 22.0, Armonk, NY: IBM Corp.

Descriptive analyses were used to characterize the sample at baseline (six to eight weeks after injury).

We used a logistic regression model to assess the predictors for RTW where we stepwise reduced the dimension. In the first step, we estimated the unadjusted model for each of the preinjury, injury-related, and postinjury factors (mentioned under Section 2.5 above) with RTW as outcome to detect all predictors with an association with RTW. In the second step, we estimated the fully adjusted model for all significant predictors from the first step. Additionally, we ensured that we have age and sex as essential properties of the cohort as well as at least one representative for each of the predictor groups (preinjury, injury-related, and postinjury) included in the model [48]. This was done to take into account potential confounding and reflect all aspects of the study in the fully adjusted model. In the third step, we estimated the final model including only the significant predictors from the fully adjusted model. The final model was developed to avoid multicollinearity, increase the power, and improve the precision (SE, CI) of the estimated odds ratios.

We used pairwise deletion for the missing data to ensure that we use all available data and achieve maximal power in the estimated models. The significance level was set to 0.05 for all analyses.

### 2.7. Ethics

The study protocol is registered in Government Clinical Trial registry, NCT00869154. The study was approved by The National Committees for Research Ethics in Norway and Norwegian Social Science Data Services, identifier NSD 20425.

## 3. Results

### 3.1. Participant Flow

**Figure 1:**
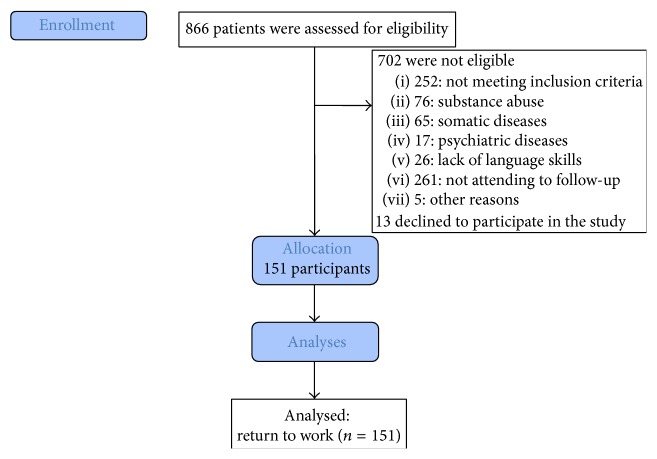
Flow diagram.

We identified 866 patients with MTBI admitted consecutively to the Department of Neurosurgery, of whom 164 patients were eligible, 13 declined to participate, and 151 patients were included in the analyses, shown in Figure 1. Of these patients, 81 were in the intervention group and 70 in the control group in the RCT.

### 3.2. Baseline Data

Table 1 lists baseline characteristics. Briefly, the median age was 32 years, and 61% of the participants were men. The majority of the injuries comprised a fall (37%). A CT scan was performed on 96% of the participants and showed intracranial injury for 27% of the patients. GCS was 15 for 74% of the participants, and 28% reported PTA for more than 1 hour. At baseline six to eight weeks after MTBI, 56% of the patients were sick-listed compared to 34% at 12 months after MTBI.

The results of the logistic regression analysis are seen in Table 2. Here, we abstain from presenting the predictors which were not included in the fully adjusted model. Age, sex, and intracranial injury as injury-related factor were included in the fully adjusted model even if they were not significant in the unadjusted model (see description of the statistical analyses above). We observed in the logistic regression model at a 5% significance level a significant association between RTW at 12 months and HAD, sick-listing variables, and GOSE. To have been sick-listed the last year before injury had the largest odds ratio (OR) 7.29 (2.6, 20.3) and being sick-listed at two months after MTBI had an OR of 6.84 (2.3, 19.9). None of the physical measures like CT findings or different measures for pain was significantly associated with RTW. We estimated a pseudo-*R* ^2^ of 0.56 (Nagelkerke) and classified 86% of the patients correctly.

## 4. Discussion

Several variables predicted RTW at 12 months in subjects with persistent PCS six to eight weeks after injury. However, in our final model, four variables contributed uniquely to RTW at 12 months, namely, having been sick-listed the last year before injury, being sick-listed at two months after MTBI, severe and moderate disability at two months (GOSE), and psychological distress (HAD).

Among preinjury variables, only having been sick-listed the last year before injury was associated with RTW at 12 months. This is partly in line with other studies that indicate that premorbidity factors such as preinjury mental health are well known as predictors for PCS, but not well documented for RTW [3, 18]. Musculoskeletal pain, depression, and anxiety are the major causes of all sick leave in Norway and were the most common diagnoses preinjury in our study [49].

Several authors have found an association between age and RTW, but Stulemeijer et al. did not [8, 12, 13, 19, 50]. One explanation may be that RTW after MTBI tends to be “U” shaped, explained by more common MTBI-related claims among younger and older age groups [50]. According to Kristman et al., this is most likely due to a “healthy worker” effect, where more susceptible workers experience injury earlier in their careers, causing them to move out of the profession, but “healthier” workers experience a lower rate of injury midcareer. On the other hand, later in life, the physical effect of age may make these “healthier” workers more susceptible to injury [50]. Both in our study and in the study by Stulemeijer et al., patients were under 55 or 60 years, respectively, excluding the oldest patients with the poorest prognosis [13]. This may explain why we found no association between RTW and age unlike other studies [8, 12].

In contrast to a recently published review, we found no association between education and RTW [3, 13]. Varying patient characteristics and different job demands between different geographic regions could be one explanation for our results [3]. However, in studies of patients from Norway with multiple severe injuries or with musculoskeletal pain, low education was a negative predictor for RTW [49, 51]. Therefore, it is less likely that geographic differences could explain the difference between our study and the previously mentioned review. Another explanation could be an overestimation of higher education in our study, as we used self-reported data, because patients may have a tendency to overestimate their formal education when they answered their questionnaires.

Studies that recruited a heterogenic MTBI population have found an association between intracranial abnormality and slower RTW [8, 52]. In our model, injury-related factors were not associated with RTW, likely because we recruited a selected group of patients with persistent symptoms six to eight weeks after MTBI. Guérin et al. highlight in their study the fact that a significant proportion of their patients had an intracranial injury (43%) at CT which might have reduced the comparability of this study with other studies [12]. Our results resemble those by Guérin et al. and Nolin and Heroux in that we found that none of the trauma severity-related variables GCS, PTA, length of hospital stay, or mechanism of injury were associated with RTW [12, 20].

It is noteworthy that there were several variables that contributed to RTW at 12 months after MTBI, including symptom burden (RPQ), posttraumatic stress (PTSS), pain and health complaints (SHC). However, only psychological distress (HAD) made a unique significant impact in our final model. Because there is a high correlation between these variables and our sample size was relatively small, care must be taken to differentiate between symptom burden and psychological distress when interpreting the findings into clinical practise. Guérin et al. and Nolin and Heroux found at long-term follow-up that the number of subjective symptoms was associated with RTW. In their studies, symptom burden and psychological distress were included in the subjective symptoms [12, 20].

We investigated several variables for pain, including headache and neck pain at admission and NRS six to eight weeks after injury for pain in the head, neck, and back. There was no significant association between these single variables and RTW. Our results are largely consistent with the findings of Guérin et al. and Nolin and Heroux. In these studies, more than six symptoms and the total number of subjective complaints were reported at follow-up as associated with RTW and not a single item [12, 20].

## 5. Strengths and Limitations

A strength of this study is the avoidance of missing follow-up data for the dependent variable RTW that could bias the results, as we used data from a national register of sick leave [53, 54]. However, using data from a national register has some limitations. The register did not contain complete information if the participant was partly or completely sick-listed. It was difficult to interpret from the register if the sick leave was a result of the MTBI. Therefore, we defined all participants independent of diagnosis, whether they were partly or completely sick-listed, as not having RTW. Participants who were either students or unemployed have to be disabled for one year before they can receive any benefits from NAV. If they then received any benefits from NAV, they were defined as being sick-listed 12 months in advance. To control for this flaw, we conducted the analyses excluding students and unemployed cases (unpublished). When excluding students and the unemployed from the analyses, we found the same predictors for RTW at 12 months with one exception, GOSE. Some reviews exclude studies that have cases of intentional MTBI as assault, because the recovery is complicated by victimisation and litigation [3]. Because we used only a subgroup of patients with MTBI, those with persistent symptoms admitted to the University Hospital, our sample size was relatively small for accurately predicting the outcome. To avoid additional reduction of our sample, we therefore included both students and patients where the cause of injury was assault in our analyses. In our study, there was no association between the cause of injury and RTW.

Only 151 out of 866 patients fulfilled the inclusion criteria for the study. We missed approximately 10% of the cases in the adjusted models due to incomplete information at baseline. Missing data could be handled with statistical imputation [48]. For this small sample, a few cases can make a difference and bias the results, even with statistical imputation if the cases are not missing at random. Therefore, we choose to not use statistical imputation in our analyses. A selection bias due to missing data cannot be excluded, but in the final model the power of the model was improved by reducing missing cases from 16 (11%) to 10 (7%). Another study limitation is the collection of clinical data on the emergency stay from the medical records, where relevant information was missing such as the intensity of acute pain. CT scan was performed by 96% of the participants, which indicates that our results regarding intracranial findings are valid. Finally, strongly correlated predictors with a correlation above 0.7 were removed due to the nonsignificance in our final model, and collinearity is only a problem in the fully adjusted model.

Further research should focus on determining which preexisting and comorbidity problems have an association with RTW. As recommended by Cancelliere et al., further research should additionally focus on the association between RTW and workplace support, social support, and the role of economic factors such as compensation after an injury [3].

Our study may have some implications for rehabilitation after MTBI. Clinically, a questionnaire is easily administrated to screen for symptom burden, psychological distress, and functional outcome approximately two months after MTBI. Early functional outcomes such as being sick-listed and disability (GOSE) at baseline six to eight weeks after MTBI together with psychological distress and a premorbidity variable, such as having been sick-listed the last year before injury, were predictors for RTW. By using these predictors for RTW, vulnerable patients could be offered treatment to improve RTW in further randomised controlled intervention studies addressed to improve vocational rehabilitation.

## 6. Conclusion

Psychological distress postinjury was the main predictor for RTW in addition to global functioning and the sick leave trajectory of the subjects. These findings have implications for clinical follow-up in MTBI and should be taken into consideration when evaluating future vocational rehabilitation models.

## Figures and Tables

**Table 1:** Demographic data and clinical characteristics at baseline 6–8 weeks after mild traumatic brain injury.

Variable	Total	*n* (%)
*Preinjury factors*		
Age, years^1^	151	32 [16, 55]
Sex, men	151	92 (61%)
Single		77 (51%)
Higher education >13 years	150	64 (43%)
Employment status	150	
Full time		112 (75%)
Part-time		5 (3%)
Unemployed		9 (6%)
Student		24 (16%)
Have been sick-listed the last year before injury	151	69 (46%)
*Injury-related factors*		
Cause of injury	151	
Traffic accident		44 (29%)
Fall		56 (37%)
Assault		27 (18%)
Sports injury and others		24 (16%)
Glasgow Coma Scale (GCS)^1,2^	151	15 [13, 15]
GCS 13		8 (5%)
GCS 14		31 (21%)
GCS 15		112 (74%)
PTA > 1 hour	142	39 (27%)
Radiological examination^2^		
Intracranial injury (CT scan)	151	41 (27%)
Skull fracture	151	22 (15%)
*Postinjury factors*		
The Rivermead Post-Concussion Symptoms Questionnaire (RPQ)		
Number of symptoms (0–16)^1^	151	8 [0, 16]
The Hospital Anxiety and Depression Scale (HAD)		
Total score (0–42)^1^	143	10 [0, 30]
HAD anxiety (0–21)^1^	143	7 [0, 19]
HAD depression (0–21)^1^	143	4 [0, 14]
Posttraumatic stress (PTSS-10)^1^	150	24 [6, 68]
Expectation of favourable outcome	149	105 (70%)
Subjective health complaints (SHC)^1^	151	10 [0, 29]
Widespread pain (numbers of painful body areas)^1^	149	2 [0, 8]
Headache (NRS)^1^	148	4 [0, 10]
Neck pain (NRS)^1^	148	4 [0, 10]
Low back pain (NRS)^1^	146	2 [0, 10]
Glasgow Outcome Scale Extended (GOSE)	149	
Severe and moderate disability (GOSE < 6)		22 (15%)
Moderate disability (GOSE = 6)		100 (67%)
Good recovery (GOSE > 6)		27 (18%)
Sick-listed at 2 months (baseline)	151	85 (56%)
Sick-listed at 12 months after injury	151	52 (34%)

^1^Median [min, max].

^2^Measured at time of injury.

**Table 2:** Logistic regression analyses of baseline data in relation to return to work after mild traumatic brain injury.

	Unadjusted models				Fully adjusted model, *N* = 135			Final model, *N* = 141		
	*N*	OR	CI (95%)	*P* value	OR	CI (95%)	*P* value	OR	CI (95%)	*P* value
*Preinjury factors*										
Age	151	1.03	(1.0, 1.1)	0.061	0.98	(0.9, 1.0)	0.410			
Sex	151	1.39	(0.7, 2.7)	0.347	0.77	(0.2, 2.8)	0.693			
Have been sick-listed the last year before injury	151	**6.90**	**(3.2, 14.8)**	**<0.001**	**8.48**	**(2.6, 27.9)**	**<0.001**	**7.29**	**(2.6, 20.3)**	**<0.001**
*Injury-related factor*										
Intracranial injury (CT scan)	151	1.38	(0.6, 3.0)	0.415	1.46	(0.4, 5.3)	0.568			
*Postinjury factors*										
Postconcussion symptoms (RPQ)	151	**1.07**	**(1.0, 1.1)**	**<0.001**	1.08	(0.9, 1.4)	0.512			
Posttraumatic stress (PTSS-10)	150	**1.06**	**(1.0, 1.1)**	**<0.001**	0.96	(0.9, 1.0)	0.331			
Anxiety and depression (HAD)	143	**1.14**	**(1.0, 1.2)**	**<0.001**	**1.16**	**(1.0, 1.3)**	**0.035**	**1.14**	**(1.1, 1.2)**	**<0.001**
Expectation of favourable outcome	149	**2.28**	**(1.1, 4.7)**	**0.026**	0.61	(0.1, 3.2)	0.554			
Subjective health complaints (SHC)	151	**1.08**	**(1.0, 1.1)**	**<0.001**	1.01	(0.9, 1.2)	0.883			
Widespread pain (numbers of painful body areas)	149	**1.35**	**(1.1, 1.7)**	**0.003**	0.83	(0.5, 1.3)	0.379			
Headache (NRS)	148	**1.20**	**(1.1, 1.4)**	**0.004**	0.90	(0.7, 1.2)	0.463			
Neck pain (NRS)	148	**1.29**	**(1.1, 1.5)**	**<0.001**	1.08	(0.8, 1.4)	0.577			
Low back pain (NRS)	146	**1.19**	**(1.1, 1.3)**	**0.005**	1.15	(0.9, 1.5)	0.280			
Glasgow Outcome Scale Extended (GOSE)	149			**0.001**			**0.017**			**0.034**
Severe and moderate disability (GOSE < 6)		**1**			**1**			**1**		
Moderate disability (GOSE = 6)		**0.32**	**(0.1, 0.8)**		**0.17**	**(0.0, 0.7)**		**0.24**	**(0.1, 0.9)**	
Good recovery (GOSE > 6)		**0.05**	**(0.0, 0.2)**		**0.03**	**(0.0, 0.5)**		**0.06**	**(0.0, 0.6)**	
Sick-listed at 2 months (baseline)	151	**7.78**	**(3.3, 18.3)**	**<0.001**	**10.16**	**(2.6, 40.0)**	**0.001**	**6.84**	**(2.3, 19.9)**	**<0.001**

Significance: *P* < 0.05 marked bold.
